# Evaluation of peripapillary choroidal thickness in patients with normal-tension glaucoma

**DOI:** 10.1186/1471-2415-12-29

**Published:** 2012-07-28

**Authors:** Kazuyuki Hirooka, Kaori Tenkumo, Atsushi Fujiwara, Tetsuya Baba, Shino Sato, Fumio Shiraga

**Affiliations:** 1 Department of Ophthalmology, Kagawa University Faculty of Medicine, 1750-1 Ikenobe, Miki, Kagawa, 761-0793, Japan

**Keywords:** Peripapillary choroidal thickness, Enhanced depth imaging optical coherence tomography, Normal-tension glaucoma

## Abstract

**Background:**

To compare peripapillary choroidal thickness measurements between normal and normal-tension glaucoma eyes.

**Methods:**

Cross-sectional comparative study. 50 normal and 52 normal-tension glaucoma subjects were enrolled in the study. Peripapillary choroidal thickness was measured with spectral-domain optical coherence tomography and enhanced depth imaging. After obtaining circular B-scans around the disc, choroidal thicknesses were calculated based on the exported segmentation values. Visual fields were measured using automated perimetry. Difference in peripapillary choroidal thickness between the normal subjects and the patients with normal-tension glaucoma was analyzed.

**Results:**

There were no significant differences in age, axial length, or refraction between the two groups. Peripapillary choroidal thickness was inversely correlated with age in both the normal (r = −0.287, P = 0.04) and normal and normal-tension glaucoma (r = −0.322, P = 0.02) groups. Peripapillary choroidal thickness of inferonasal (125 vs 148 μm, *P <* 0.05), inferior (101 vs 122 μm, *P <* 0.05), or inferotemporal (100 vs 127 μm, *P <* 0.05) regions were significantly thinner in the normal-tension glaucoma group as compared to normal subjects. Superior visual hemifield defect was significantly worse than inferior visual hemifield defect in normal and normal-tension glaucoma patients.

**Conclusion:**

As compared to normal subjects, peripapillary choroidal thickness was significantly thinner in the normal and normal-tension glaucoma patients, at least in some locations.

## Background

Normal-tension glaucoma (NTG) accounts for 92% of primary open-angle glaucoma (POAG) in Japanese patients [1]. This clinical term is often used to describe patients with open-angle glaucoma (OAG) in whom the measured untreated intraocular pressure (IOP) is always within a statistically normal range. Multicenter clinical trials have confirmed the value of reducing the IOP in POAG [2,3] and NTG [4,5] patients. There has yet to be a consensus regarding the specific relationship between the IOP and NTG. However, elevated IOPs are not always associated with glaucomatous optic neuropathy (GON), and in many cases, a progression of the GON has been observed after lowering the IOP. Thus, these findings suggest that in addition to the presence of an elevated IOP, there may be other factors that play an important role in glaucoma pathogenesis. After the original anatomical findings of peripapillary chorioretinal atrophy were first reported [6], many authors speculated that the ciliary circulation, particularly the choroidal supply to the papillary area, could be an etiological factor in glaucoma [7,8].

Abnormal choroidal blood supply has been suggested to be one factor responsible for the occurrence of GON in OAG patients [7]. Yin et al. [9] found that POAG eyes had the thinnest choroid both globally and in the peripapillary region. Recently, there has been an increasing interest in determining the specific role that the choroid plays in the pathogenesis of OAG. In general, there is a depth-dependent roll-off in the sensitivity of the spectral-domain optical coherence tomography (OCT) instruments [10,11]. Due to these potential problems, this led to the development of enhanced depth imaging (EDI) spectral-domain OCT, which subsequently made it possible to perform in vivo cross-sectional imaging of the choroid [10]. Other investigators have reported that the measurement of choroidal thickness with the use of OCT at a longer wavelength [12,13]. Through use of this new methodology, investigators have been able to examine the choroidal thickness in glaucoma patients [11-15]. While some of these investigations have reported finding that the choroidal thickness did not seem to differ between normal and glaucoma patients [14-17], Usui et al. [17] have reported that the choroidal thickness in highly myopic NTG was significantly thinner as compared to controls. However, this latter study only included spherical equivalent refractive errors between −6 and −12 diopters and axial lengths that exceeded 26.5 mm.

We recently showed that the choroidal thickness at 3 mm nasal from the fovea was significantly thinner in NTG eyes as compared to normal subjects and also correlated with the mean deviation slope [18]. However, choroidal thickness was measured in only one peripapillary location. The purpose of the current study was to determine if there were differences in the EDI OCT peripapillary choroidal thickness measurements obtained in normal and NTG eyes.

## Methods

Consecutive NTG patients who visited the Kagawa University Hospital, Kagawa, Japan between May 2011 and August 2011 were recruited for the study. One eye per subject was randomly selected for the study. Inclusion criteria included a cylinder within *±* 2.0 D. In order to be enrolled, subjects were required to have had no previous ocular surgery. Subjects were excluded if they had any history of retinal diseases (e.g., diabetic retinopathy, macular degeneration, retinal detachment) or laser therapy, had a poor image quality because of unstable fixation, or had severe cataract. Exclusion criteria also included any history of treatment with medications that could affect retinal thickness, such as intravitreal anti-VEGF therapy. The same examiner performed the EDI OCT examinations in all cases in the morning. All subjects underwent visual acuity, refraction, central and peripheral fields, slit lamp, and gonioscopy examinations. All NTG patients had typical glaucomatous optic disc damage with nerve fiber bundle defects and open angle. NTG was diagnosed when there was an untreated peak IOP of ≤ 21 mmHg, which included the 24-hour fluctuation measurements. Glaucomatous eyes were defined as eyes exhibiting structural glaucomatous changes (vertical cup-disc asymmetry between fellow eyes of ≥ 0.2, a cup-to-disc ratio of ≥ 0.6, and neuroretinal rim narrowing, notches, localized pallor, or retinal nerve fiber layer defects with glaucomatous visual field (VF) loss in the corresponding hemifield). Glaucomatous VF defects were defined as defects with consecutive, repeated abnormal standard automated perimetry results (≥ 2 contiguous points with a sensitivity loss of *P <* 0.05 in the superior or inferior arcuate areas, a 10 dB difference across the nasal horizontal midline at > 2 adjacent locations, or an abnormal glaucoma hemifield test result). Other inclusion criteria for the normal subjects included an IOP ≤ 21 mmHg and a normal ophthalmoscopic appearance of the optic nerve. All eligible subjects received a detailed explanation about the study and signed an informed consent form in accordance with the principles embodied in the Declaration of Helsinki. This study was approved by the institutional review board at the Kagawa University Hospital.

**Figure 1:**
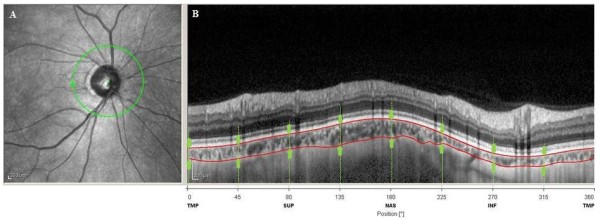
**The location of the scan used (A) and images from the 360° peripapillary EDI OCT scans (B).** Peripapillary choroidal thickness (between up-pointing arrow and down-pointing arrow) was measured from the outer border of the retinal pigment epithelium (RPE) to the inner border of the sclera (arrowheads).

The examination protocol was conducted in a seated, resting position and included measurements of the axial length and keratometry in both eyes using an IOLMaster (Carl Zeiss Meditec, Dublin, CA). Subsequently spectral domain OCT (SD-OCT) scans of the peripapillary regions were performed using the Heidelberg Spectralis (Heidelberg Engineering, Heidelberg, Germany). The SD-OCT images were obtained by using enhanced depth imaging. With this method, the focus is manually placed more posteriorly than would normally be done during standard retinal SD-OCT imaging, as this improves the resolution of the choroidal detail. However, because of this, the images that are produced are inverted. The peripapillary region was scanned using a 360°, 3.4 mm diameter circle scan that was centered on the optic disc (Figure 1A). A reader who was masked to the clinical patient data used the provided Heidelberg Eye Explorer software (version 1.5.12.0; Heidelberg Engineering) to manually delineated the choroidal thickness. This thickness was defined as the area of visible choroidal vasculature between the outer retinal pigment epithelial border and the inner scleral wall (Figure 1B). Subsequently, the Humphrey central 30–2 full-threshold program (C30-2 program) was used to determine the visual field, which was defined as reliable when fixation losses, false-positive errors, and false-negative errors were less than 20%.

Statistical analysis was performed using SPSS for Windows (SPSS Inc., Chicago, IL). Differences for age, IOP, axial length, refraction and visual hemifield defects were analyzed using an independent Student’s *t*-test. The Shapiro-Wilk test was used to determine the normality of the distribution of the choroidal thickness data. The choice of the Mann–Whitney *U* or independent Student’s *t*-test to study the significance of difference was based on the presence or absence of the normality of distribution. Gender differences were analyzed by Fisher’s exact probability test. The correlation between the axial length, age, refraction, retinal nerve fiber layer (RNFL) thickness or mean deviation (MD) and the choroidal thickness was analyzed using a Spearman’s rank correlation coefficient test. A *P* value less than 0.05 was considered statistically significant. The data are presented as mean *±* standard deviation (SD).

## Results

A total of 103 subjects (50 normal subjects and 52 NTG patients) met the eligibility criteria. There were no significant differences noted between normal and NTG groups for age (62.4 vs 66.2 years, *P =* 0.10), gender (*P* = 0.12), axial length (24.4 vs 24.9 mm, *P* = 0.10), refraction (−2.2 vs −2.6 diopter, *P* = 0.57), or corneal refraction (43.8 vs 43.4 diopter, *P* = 0.15) (Table 1). However, the IOP in the NTG group was significantly lower than that observed in the normal subjects (14.1 vs 12.8 mmHg, *P* = 0.004).

**Table 1:** Clinical characteristics of the study participants

	**Normal**	**NTG**	**P value**
N	50	52	
Age (y)	62.4 *±* 10.0	66.2 *±* 13.1	0.10
Gender (M/F)	28/22	21/31	0.12
Axial length (mm)	24.4 *±* 1.4	24.9 *±* 1.5	0.10
IOP (mmHg)	14.1 *±* 2.8	12.8 *±* 1.9	0.004
MD (dB)		-12.1 *±* 7.3	
Refraction (D)	-2.2 *±* 4.0	-2.6 *±* 3.7	0.57
Corneal refraction (D)	43.8 *±* 1.1	43.4 *±* 1.3	0.15

***NTG*, normal-tension glaucoma; *M*, male; *F*, female; **

***IOP*, intraocular pressure; *MD*, mean deviation; *D*, diopter.**

**Figure 2:**
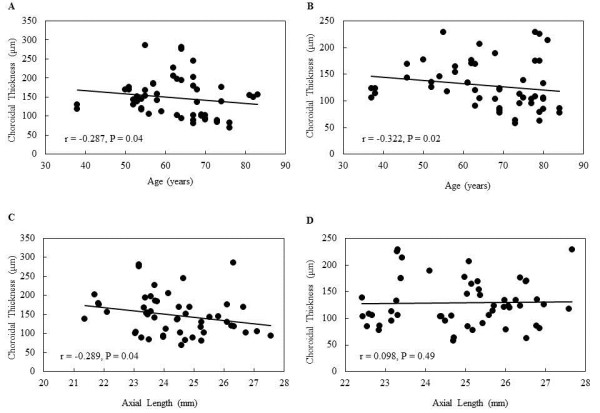
**Scatter plot of the simple linear regression analysis between the age and (A) the mean peripapillary choroidal thickness in normal subjects, and (B) the mean peripapillary choroidal thickness in patients with normal-tension glaucoma (NTG).** Scatter plot of the simple linear regression analysis between the axial length and (**C**) the mean peripapillary choroidal thickness in normal subjects, and (**D**) the mean peripapillary choroidal thickness in patients with NTG.

Mean peripapillary choroidal thicknesses were 148.8 *±* 53.3 μm and 128.1 *±* 44.6 μm in the normal and NTG subjects, respectively (*P* = 0.04) (Table 2). Peripapillary choroidal thicknesses of the inferonasal (*P* = 0.04), inferior (*P* = 0.03), or inferotemporal (*P* = 0.01) areas in the NTG group were significantly thinner than those observed in the normal subjects (Table 2). In normal subjects, age was correlated with the superonasal (r = −0.322, *P* = 0.02), nasal (r = −0.415, *P* = .003), inferonasal (r = −0.347, *P* = 0.01), inferior (r = −0.291, *P* = 0.04) and average (r = −0.287, *P* = 0.04) (Figure 2A) choroidal thicknesses. In NTG subjects, age was correlated with superior (r = −0.307, *P* = 0.03), superonasal (r = −0.339, *P* = 0.01), nasal (r = −0.402, *P* = 0.003), inferonasal (r = −0.371, *P* = 0.006), inferior (r = −0.271, *P* = 0.049) and average (r = −0.322, *P* = 0.02) (Figure 2B) choroidal thicknesses. In the normal subjects, axial length was correlated with temporal (r = −0.438, *P* = 0.001), superotemporal (r = −0.346, *P* = 0.01), inferotemporal (r = −0.334, *P* = 0.02) and average (r = −0.289, *P* = 0.04) (Figure 2C) choroidal thicknesses. In the normal subjects, the refraction was only correlated with the temporal choroidal thickness (r = 0.346, *P* = 0.01). In the NTG subjects, none of the measurements were correlated with either the axial length (Figure 2D) or refraction (all *P* > 0.05). There was also no correlation noted between the IOP and the choroidal thickness (all *P* > 0.05). Spearman’s rank correlation coefficient test demonstrated that RNFL thickness and peripapillary choroidal thickness measurements were not significantly correlated for any peripapillary location (r *<* 0.102, *P* > 0.21), except superior (r = 0.358, *P* = 0.02) (Table 3). There was no significantly correlation between MD and mean peripapillary choroidal thickness (r = 0.188, *P* = 0.18).

**Table 2:** Choroidal thickness in normal and NTG eyes

**Location**	**Choroidal thickness (μm)**		**P value**
	**Normal**	**NTG**	
Temporal	142.6 *±* 69.1	129.3 *±* 55.9	0.29
Superotemporal	149.6 *±* 62.2	131.5 *±* 51.1	0.11
Superior	163.0 *±* 56.1	143.1 *±* 51.5	0.07
Superonasal	169.8 *±* 57.5	148.5 *±* 53.5	0.06
Nasal	168.5 *±* 59.4	146.4 *±* 55.1	0.05
Inferonasal	148.2 *±* 60.3	125.0 *±* 49.3	0.04
Inferior	121.8 *±* 55.5	100.8 *±* 37.0	0.03
Inferotemporal	127.1 *±* 60.7	100.0 *±* 42.7	0.01
Average	148.8 *±* 53.3	128.1 *±* 44.6	0.04

***NTG*, normal-tension glaucoma.**

**Table 3:** Sperman's rank correlation between RNFL thickness and choroidal thickness by peripapillary location

**Location**	**Sperman's rank**	***P *value**
	**correlation coefficient (r)**	
Temporal	-0.076	0.63
Superotemporal	0.037	0.81
Superior	0.358	0.02
Superonasal	0.011	0.95
Nasal	0.085	0.59
Inferonasal	-0.191	0.21
Inferior	-0.052	0.74
Inferotemporal	0.102	0.47

***RNFL*, retinal nerve fiber layer**

Mean total deviation of the inferior visual hemifield was −10.4 *±* 8.0 dB, while mean total deviation of the superior visual hemifield was −12.9 *±* 8.1 dB (Table 4). There was a significant difference between the inferior and superior visual hemifield defects (*P* = 0.04).

**Table 4:** Visual hemifield defects in the NTG patients

**Superior**	**Inferior**	**P value**
-12.9 *±* 8.1 dB	-10.4 *±* 8.0 dB	0.04

***NTG*, normal-tension glaucoma.**

## Discussion

When compared to the eyes of the normal subject, the NTG eyes had a significantly thinner mean peripapillary choroidal thickness. Although both age and axial length were correlated with the mean peripapillary choroidal thickness in the normal subjects, only age was correlated with the mean peripapillary choroidal thickness in the NTG patients. The inferior peripapillary choroidal thickness was also thinner in the NTG patients as compared to the control subjects. Interestingly, the superior visual hemifield defect was worse than the inferior visual hemifield defect in the NTG patients.

Previous studies that performed histological analyses of glaucomatous eyes have reported finding both increases and decreases in the choroidal thicknesses [12,19,20]. However, since fixation of the choroid before histologic analysis causes shrinkage, this will affect the thickness measurements. As such, histological analysis can only provide a rough estimate of the in vivo choroid thickness. Mwanza et al. [16] recently examined EDI OCT measurements of the choroidal thickness and reported finding no differences between the normal, NTG, and POAG subjects. However, the macular choroidal thickness was only measured in 38 normal and 20 NTG subjects, which was smaller than our current study. Usui et al. [17] examined subjects using high-penetration OCT and showed that the choroidal thickness was significantly thinner in high myopic NTG patients as compared to high myopic controls. They concluded that this choroidal thinning was related to the highly myopic NTG.

Generalized choroidal thinning is associated with vessel loss, and occurs predominantly in the inner choroids [12]. It is possible that this phenomenon could reduce the blood flow volume in the low-pressure choroidal bed. Previous evidence has shown that the peripapillary choroid is responsible for supplying the prelaminar region, with the most convincing evidence for this derived from fluorescence angiographic studies [21,22]. However, there are other postmortem morphological studies that have reported evidence that strongly supports the theory that the peripapillary choroid does not supply the prelaminar region of the optic nerve head [23-26]. However, in the Cioffi & Van Buskirk study, [26] they did find that some branches of the circle of Haller and Zinn (CHZ) and the short posterior ciliary arteries (PCAs) coursed through the choroid and ultimately supplied the prelaminar region. Hayreh [27] further discussed this discrepancy and pointed out that once the short and long PCAs and the branches of the CHZ had pierced the sclera, they then essentially became a part of the choroid and were no longer considered to be branches of the PCAs. Thus, the apparent confusion and conflicting speculations appear to be simply a matter of semantics.

There are potential limitations in our study. Although choroidal thickness seems to be influenced by diastolic blood pressure [15], there was no data on the blood pressure in the two groups of patients. Second, the current operating software of Heidelberg Spectralis OCT does not provide automatic segmentation of the choroid. However, Yamashita et al. [28] recently reported that intraobserver intraclass correlation coefficients (ICCs) for subfoveal choroidal thickness was 0.976 (*P <* 0.001). The measurements were extremely reproducible. Third, there seems to be significant diurnal variation of choroidal thickness [29,30]. The choroidal thickness decreased from 9:00 AM through the time point at 11:00 AM [30]. The OCT scans were performed in the morning in our study.

## Conclusion

In conclusion, our current study demonstrated that the mean peripapillary choroidal thickness was significantly thinner in the NTG eyes when compared to normal subjects. In the NTG patients, the peripapillary choroidal thickness of the inferonasal, inferior, or inferotemporal regions decreased in conjunction with a corresponding worsening of the visual hemifield defect.

## Competing interests

The authors declare that they have no competing interests.

## Authors’ contributions

KH Conception and design. AF Acquisition of data. KH Analysis and interpretation of data. KH, KT Preparation. KH, FS Review. KH, KT, AF, TB, SS, FS Approval of manuscript. All authors read and approved the final manuscript.

## Pre-publication history

The pre-publication history for this paper can be accessed here:

http://www.biomedcentral.com/1471-2415/12/29/prepub
